# Selection index for economically important traits in Boer crossbred goats using principal component analysis

**DOI:** 10.1371/journal.pone.0310841

**Published:** 2025-04-01

**Authors:** Zeleke Tesema, Belay Deribe, Mekonnen Tilahun, Alemu Kefale, Mesfin Lakew, Tesfaye Getachew, Getachew Worku Alebachew, Solomon Gizaw

**Affiliations:** 1 Debre Birhan Agricultural Research Center, Debre Birhan, Ethiopia; 2 Sirinka Agricultural Research Center, Woldia, Ethiopia; 3 Andasa Livestock Research Center, Bahir Dar, Ethiopia; 4 Amhara Agricultural Research Institute, Bahir Dar, Ethiopia; 5 International Center for Agricultural Research in the Dry Areas (ICARDA), Addis Ababa, Ethiopia; 6 International Livestock Research Institute (ILRI), Addis Ababa, Ethiopia; Ain Shams University Faculty of Agriculture, EGYPT

## Abstract

The optimal strategy for genetic selection is a selection index based on economic weight; however, in developing countries where economic weight estimation is not always evident and easy for breeders due to a lack of economic data. Thus, this study aimed to construct selection indices for crossbred goats, which could be used as an alternative to economic selection index and to explore the relationship among economically important traits. The data set contained records of birth weight (BW), weaning weight (WW), pre-weaning weight gain (ADG), pre-weaning Kleiber ratio (KR), pre-weaning relative growth rate (RGR), pre-weaning growth efficiency (GE), and pre-weaning survival (RR) of crossbred goats. Genetic parameter estimates were obtained using a single-trait animal model. General linear model, principal component analysis, and cluster procedures of SAS were also used for data analysis. Kid survival was negatively correlated with all investigated traits except BW. Traits such as KR, GE, RGR, WW, and ADG were highly and positively correlated. According to the Kaiser method, two principal components were selected from seven investigated traits. The first principal component (PC1) explained 57.71%, and the second principal component (PC2) explained 14.57% of the estimated breeding value variance, totaling 72.28% of the total genetic additive variance. PC1 explained most of the direct additive genetic variation and correlated with the estimated breeding value of WW, ADG, KR, GE, and RGR, whereas PC2 was correlated with the estimated breeding value of BW and RR. Besides, the cluster analysis categorized seven traits into two major groups. The first group includes BW and RR, whereas traits such as WW, ADG, KR, GE, and RGR were included in the second group. Therefore, two based selection indices, or principal component scores (PCS) were derived. Animals with higher PCS1 could be used to improve WW, ADG, KR, GE, and RGR, whereas animals with higher PCS2 scores could be used to improve BW and pre-weaning survival of crossbred kids. The selection of the most appropriate and specific selection index regarding the two groups of traits is determined by the breeding objectives defined for specific genetic improvement program. These selection indices could be used as an alternative approach when economic weights for traits of interests are not available to construct the economic selection index. However, further works should be done on refining the selection indices and validating them in independent datasets.

## Introduction

Goats are the source of a variety of products and services for smallholder farmers. The crossing of indigenous goats with Boer goats was initiated in 2007 in Ethiopia to enhance their productivity. Beyond the crossing, its combination with selection could enhance genetic gain [1]. Among production traits, pre-weaning growth traits had considerable genetic and phenotypic correlations with growth performance, meat production, reproduction, health, and other productive traits of small ruminants [2–6]. Besides, goat producers sold most Boer crossbred kids around six months of age [7]. Therefore, improving the growth rate until the market weight could enhance the productive efficiency in such a situation. According to previous studies [8,9], improvements in growth and meat production efficiency could be made through selection for efficiency-related traits such as growth efficiency, relative growth rate, and Kleiber ratio besides direct selection based on live weight of animals. Thus, the selection of animals for these important traits has deterministic effects on the profitability of goat production enterprises.

Most genetic improvement programs define multi-trait breeding goals, which require the construction of selection indices for simultaneous improvement of the traits of interest. The breeding values for each trait of interest were weighed by economic weights in the aggregate genotype [10]. Multiple trait selection using selection indices improves both the economic and breeding values and thus can be considered the fastest and most efficient manner to improve the aggregate breeding value [10,11]. Hence, applying efficient selection indices by defining the economic value of the important traits is of great necessity. The economic weight of traits could be estimated using partial budget analysis, choice experiments by analyzing farmers’ willingness to pay, and bio-economic modeling [12]. However, the economic weight associated with each genetic value in selection indices is empirically attributed due to illiteracy, lack of record-keeping and small flock sizes [13]. Thus, making the selection more effective without empirical considerations is important to enhance the genetic gain.

Deriving alternative and applicable selection indices allow goat producers to make balanced selection decisions and identify the most profitable animal, taking into account each animal's relevant production and fitness traits. Principal component analysis (PCA) based on the estimated breeding value of several traits is used to explore the genetic relationships among the traits. It can be considered to be genetic selection indices [14,15] since standardized score coefficients are linear combinations of all predicted genetic values for traits [14,16]. PCA accounts for trait correlations and may weight each trait using its eigenvector and contribution to the overall explained variance; it constructs more realistic selection indices [17,18]. Thus, PCA would facilitate the simultaneous selection of the traits of interest [14,19]. The optimal strategy for genetic selection is a selection index based on economic weight; however, in developing countries where economic weight estimation is not always evident and easy for breeders due to a lack of economic data, the PCA is an objective method to weight features [20]. Therefore, this study aimed to explore the relationship among pre-weaning growth, efficiency-related traits, and survival; to identify trait(s) that could be used as selection criteria, and to construct selection indices for Boer crossbred goats using principal component analysis.

## Material and methods

### Ethics statement

This study was based on data collected from live goats managed at Sirinka Agricultural Research Center sheep and goat breeding station without any invasive procedure through close monitoring of researchers. Anesthesia, euthanasia, or animal sacrifice was not part of the study. In addition, animal handling, data collection formats and procedures were reviewed and approved by Amhara Agricultural Research Institute, Ethiopia in the annual research review forum.

### Data set description

The pedigree and phenotype data of Boer x Central Highland goats were obtained from Sirinka Agricultural Research Center sheep and goat breeding station in northeastern Ethiopia. The breeding station is located at an altitude of 1850 m.a.s.l and 11°45’ 00” N and 39°36’ 36” E. The area receives about 950 mm of annual rainfall on average. The area has a moderately warm climate, with average daily temperatures ranging from 13.7 to 26.4 °C. The data were collected by researchers under on-station management of animals. Goats were managed semi-intensively, i.e., allowed to graze for about six hours per day on a natural pasture and supplemented with 0.10–0.40 kg of concentrate mixture consisting of wheat bran, *Noug* seed cake, and salt, based on their physiology, sex, and age. They were housed in semi-open concrete barns at night based on age, physiology, and sex.

The kids were weaned after approximately 90 days of age. The investigated traits were birth weight (BW), weaning weight (WW), pre-weaning weight gain (ADG), pre-weaning Kleiber ratio (KR), pre-weaning growth efficiency (GE), pre-weaning relative growth rate (RGR), and pre-weaning survival (RR) of kids. Efficiency-related traits were computed as follow: GE =  (WW – BW)/BW x 100, RGR =  (ln (WW) – ln (BW)) / 90 days x 100, and KR =  ADG/WW^0.75^. The detailed descriptions of a data structure are summarized in Table 1.

**Table 1:** Description of the data structure for early growth, survival, and efficiency-related traits in Boer x Central Highland goats.

Item	Trait						
	BW	WW	ADG	KR	GE	RGR	RR
Number of records	875	638	638	638	674	674	831
Number of sires	25	22	22	22	22	22	26
Number of dams	238	209	209	209	219	238	239
NPR/ sire	35	29	29	29	30.6	30.6	32
NPR/ dam	4	3	3	3	3.07	3.07	3
Mean	2.52	9.80	80.1	13.99	293.5	1.46	–
SD	0.57	3.39	35.0	2.12	131.4	0.36	–
CV (%)	19.2	28.7	37.4	14.2	39.0	37.1	–

BW, birth weight; WW, weaning weight; ADG, pre-weaning weight gain; KR, pre-weaning Kleiber ratio; RGR, pre-weaning relative growth rate; RR, pre-weaning survival; SD, standard deviation; CV, coefficient of variation; NPR, number of progeny with records.

### Data analysis

All possible systematic factors, which may affect the performance of animals were considered in this study. The systematic effects included in the model were the kid's birth type (single and multiple births), sex of the kid (male and female), the season of birth (dry, short, and main rain), parity of doe (1, 2, 3, 4, and ≥ 5), year of birth (2009, 2010, …, 2018), dam genotype (local, F1, and F2), Boer blood level (25, 50, 62.5, and 75%), and kid genotype (F1, F2, and F3). The significance of systematic effects was evaluated using the GLM procedure of SAS [21], which is suitable to analyze unbalanced breeding data. After evaluating the significance of  systematic effects, genetic parameter estimates for a continuous data type (growth and efficiency-related traits) were derived by the AI-REML algorithm using WOMBAT software [22]. However, the Bernoulli distribution is typically selected when the response data are binary (only taking on the values 0 and 1). The appropriate link for this type of data is logistic regression or logit link. Thus, the genetic parameter estimates for survival were derived using the AI-REML method after logit transformation of the data with a linear mixed model using ASREML software [23] fitting animal model. By including and excluding the maternal genetic effect and maternal permanent environmental effect, six models were evaluated for each trait, and the matrix representation of the selected models as per the log-likelihood ratio test is presented in Table 2.

**Table 2:** Selected model for each trait.

Traits	Selected model
BW	**y = Xβ + Z**_**1**_**a + Z**_**2**_**m + ε** with Cov (**a, m**) = 0
WW	**y = Xβ + Z**_**1**_**a + Z**_**2**_**m + ε** with Cov (**a, m**) = **A**σ_am_
ADG	**y = Xβ + Z**_**1**_**a + Z**_**2**_**m + Z**_**3**_**c + ε** with Cov (**a, m**) = **A**σ_am_
KR	**y = Xβ + Z**_**1**_ **a + Z**_**2**_ **c + ε**
GE	**y = Xβ + Z**_**1**_**a + Z**_**2**_**m + ε** with Cov (**a, m**) = 0
RGR	**y = Xβ + Z**_**1**_**a + Z**_**2**_**m + Z**_**3**_**c + ε** with Cov (**a, m**) = **A**σ_am_
RR	**y = Xβ + Z**_**1**_ **a + Z**_**2**_ **c + ε**

Where: **y** is the vector of the records of traits; **b, a, m, c,** and **ε** are vectors of fixed effects, additive direct genetic, maternal additive genetic, maternal permanent environmental effects and residual effects, respectively; **X, Z**_**1**_**, Z**_**2,**_ and **Z**_**3**_ are incidence matrices that relate these effects to the records. It was assumed that **a, m, c,** and **ε** are normally distributed with the mean zero and variance **Aσ**^**2**^_**a**_**, Aσ**^**2**^_**m**_**, I**_**p**_**c**^**2**^, and **I**_**n**_**ε**^**2**^*,* respectively*.* Where **A** is the numerator relationship matrix between animals**; σ**_**am**_ is the covariance between additive direct and maternal genetic effects**; I**_p_ and **I**_n_ are identity matrices with orders equal to the number of does and kids, respectively. **σ**^**2**^_**a,**_ **σ**^**2**^_**m,**_ **c**^**2,**^ and **ε**^**2**^ are the direct additive genetic variance, maternal additive genetic variance, maternal permanent environmental variance, and residual variance, respectively.

A principal component analysis is a multivariate statistical method that reduces a set of observable variables and accounts for the greatest variance using the fewest possible composite variables. However, each variable had its contribution to the total variance [24]. The Kaiser-Meyer-Olkin measures of sampling adequacy and Bartlett's test of sphericity were computed first to verify the accuracy of the principal component analysis of the data sets. The appropriateness of the PCA was further tested using communalities and ratio of cases to variables. The PROC PRINCOMP function of SAS [21] was used to conduct the principal component analysis following these tests. The number of principal components that explain the maximum genetic variation in the data was determined according to Kaiser [25]. The estimated breeding values (EBV) analyzed were those for birth weight, weaning weight, weight gain, Kleiber ratio, growth efficiency, relative growth rate, and kids’ survival.

Cluster analysis was used to represent the grouping of traits [26], and the PROC CLUSTER procedure of the SAS program [21] using the hierarchical clustering method was used for cluster analysis. A complete linkage method, which takes the farthest distance, was used for cluster analysis.

The following procedures were followed to construct the PCA-based selection indices. To avoid inconsistent solutions in the principal components due to the differences in scales and the magnitude of the variables, the EBV was standardized to zero mean and unit variance according to Boligon et al. [16]:


$$Zi=\frac{\left(Xi-\bar{X}\right)}{\delta }$$

where Z_i_ is the standardized value of X_i_ variable, $\bar{X}$ is the mean of the i^th^ trait, and *δ*_i_ is the corresponding standard deviation.

The standardized score coefficients of each EBV in each PC were obtained as follows [14]:


$$\frac{eigenvector\text{ij}}{\sqrt{eigenvalue}\text{j}}$$

where V_ij_ is the standardized score coefficient for EBVs of the i^th^ trait in the j^th^ principal component, the eigenvalue of the PC is connected with the variance of all EBVs of traits involved in the PC, which intern constitutes an eigenvector [27]. The absolute value of these eigenvectors explains the importance of traits in a PC.

Using the standardized estimated breeding value, each principal component can generate a new value, called a principal component score. The principal component scores (PCS) were calculated as follows:


$$PCSjk=\overset{m}{\underset{i=1}{\sum }}Vij\times SEBVik$$

where PCS_*jk*_ is the principal component score for the *k*^th^ animal in the *j*^th^ principal component, V_*ij*_ is the standardized score coefficient for the estimated breeding value of the *i*^th^ trait in the j^th^ principal component, and SEBV_ik_ is the standardized estimated breeding value of *i*^*th*^ trait for the *k*^th^ animal.

## Results

### Correlation estimates based on standardized EBV

The correlations among investigated traits based on the standardized estimated breeding value are presented in Table 3. The genetic correlation of birth weight with all traits except survival was low and negative, as indicated in Table 3. Likewise, survival was negatively correlated with all investigated traits except birth weight. Kleiber ratio, growth efficiency, relative growth rate, weaning weight, and pre-weaning weight gain were highly and positively correlated.

**Table 3:** Correlation matrix and heritability estimates (the diagonal) for investigated traits.

	BW	WW	ADG	KR	GE	RGR	RR
BW	0.31^a^;0.38^b^						
WW	−0.041	0.50^a^;0.12^b^					
ADG	−0.047	0.806	0.57^a^;0.09^b^				
KR	−0.061	0.612	0.923	0.18^a^;0.18^b^			
GE	−0.064	0.550	0.739	0.809	0.001^a^;0.054^b^		
RGR	−0.065	0.441	0.795	0.945	0.879	0.23^a^;0.075^b^	
RR	0.258	−0.097	−0.084	−0.056	−0.027	−0.032	0.006^a^;0.006^b^

BW, birth weight; WW, weaning weight; ADG, pre-weaning weight gain; KR, pre-weaning Kleiber ratio; RGR, pre-weaning relative growth rate; RR, survival.

^a^=direct heritability estimate,

^b^=total heritability estimates [6,7,28].

### Principal component analysis

In this study, the estimate of Kaiser-Meyer-Olkin, which is a measure of sampling adequacy was 0.673. Bartlett's test of sphericity was used to determine whether the correlation matrix was significant for all traits investigated, and the results were significant (chi-squared value: 5143.5, P <  0.0001). According to Kaiser [25], i.e., principal components with eigenvalues greater than one, two principal components were selected from seven PCs in this study. Eigenvalues of factors indicate the contribution of each component to total variation. The first principal component (PC1) explained 57.71%, and the second principal component (PC2) explained 14.57% of the breeding value variance, totaling 72.28% of the total genetic additive variance (Table 4). Thus, two components were sufficient to explain most of the variation among genetic values estimated for the investigated traits.

**Table 4:** Eigenvalues and variance proportions for the principal components of the standardized breeding values.

Principal components	Eigenvalue	Variance	Cumulative variance
PC1	4.039	0.577	0.577
PC2	1.020	0.146	0.723
PC3	0.984	0.141	0.863
PC4	0.663	0.095	0.958
PC5	0.240	0.034	0.992
PC6	0.043	0.006	0.998
PC7	0.011	0.002	1.000

PC, principal component.

**Fig 1:**
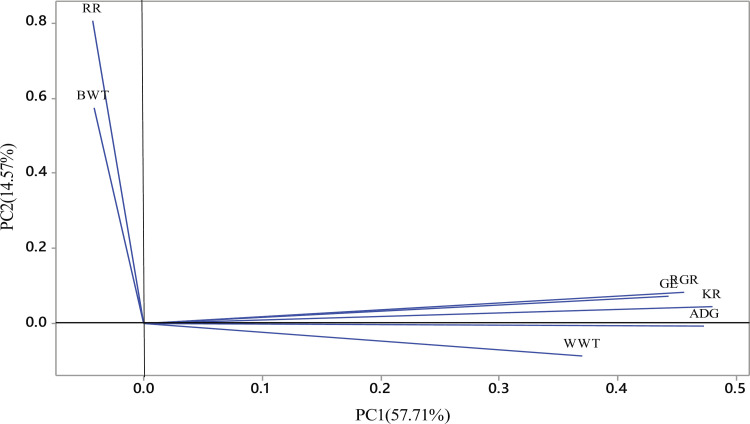
Loading plot for investigated traits.

The degree to which variables and components correlated reflects the relative importance of the variables. PC1 explained most of the direct additive genetic variation, and it correlated with the estimated breeding values of weaning weight, weight gain, Kleiber ratio, growth efficiency, and relative growth rate, whereas PC2 was correlated with estimated breeding values of birth weight and survival of kids (Table 5, Fig 1). The selection of animals could be based on only two components rather than all attributes, according to the positive connection between principal components and estimated breeding values of traits. Birth weight and survival were negatively correlated with PC1, which accounted for 57.71% of the total variation while weaning weight and weight gain were negatively associated with PC2. The communalities of investigated traits in this study are shown in Table 5. The variation in features that can be explained by a common component is called communality, and its value is ranged between 0 and 1. The communality varied from 0.344 for BW to 0.933 for KR. The loading plot for investigated traits is shown in Fig 1.

**Table 5:** Correlation coefficients between standardized breeding values of the studied traits with two principal components and communalities (h^2^) of traits.

Trait	PC1	PC2	Communalities
BW	−0.042	0.575	0.344
WW	0.371	−0.084	0.562
ADG	0.473	−0.006	0.904
KR	0.473	0.046	0.933
GE	0.443	0.072	0.799
RGR	0.456	0.084	0.849
RR	−0.043	0.805	0.669

BW, birth weight; WW, weaning weight; ADG, pre-weaning weight gain; KR, pre-weaning Kleiber ratio; RGR, pre-weaning relative growth rate; RR, survival.

### Cluster analysis

**Fig 2:**
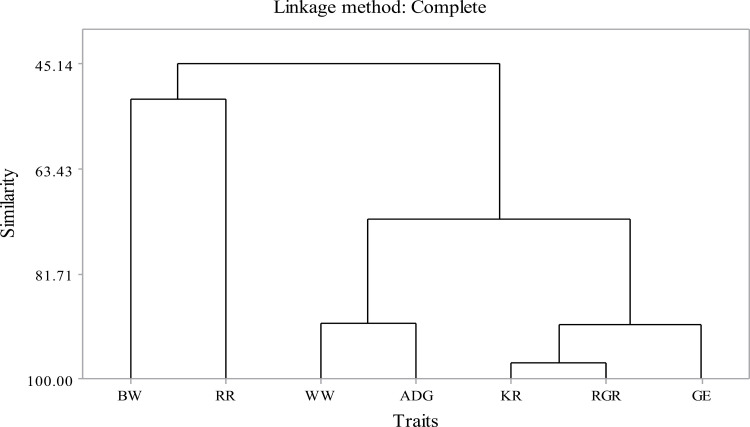
Dendrogram based on complete linkage methods for traits investigated.

Fig 2 displays the cluster analysis for the analyzed traits using the complete linkage approach. Seven traits were categorized into two major groups. The first group includes birth weight and pre-weaning survival. Traits such as weaning weight, weight gain, Kleiber ratio, growth efficiency, and relative growth rate were included in the second group.

### Selection indices

The standardized score coefficients of each standardized estimated breeding value for all traits in two PCs are shown in Table 6. The weight of each trait in each PC was different. Traits such as WW, ADG, KR, RGR, and GE had good weight in the first principal component, while BW and RR had good weight in the second principal component. Using these standardized score coefficients for each trait in both PCs, the principal component scores (PCS), which could be used as a selection index for each animal in the first two principal components were calculated as follows:

**Table 6:** Standardized score coefficients (V) of each standardized estimated breeding value for all traits in two PCs.

Trait	V-PC1	V-PC2
BWT	−0.021	0.569
WWT	0.184	−0.083
ADG	0.235	−0.006
KR	0.235	0.045
GE	0.221	0.071
RGR	0.227	0.083
RR	−0.021	0.797

BW, birth weight; WW, weaning weight; ADG, pre-weaning weight gain; KR, pre-weaning Kleiber ratio; RGR, pre-weaning relative growth rate; RR, survival.


$$\begin{array}{l} PCS1=–0.021EBV_{BW}+0.184EBV_{WW}+0.235EBV_{ADG}+0.235EBV_{KR}+0.221EBV_{GE}+\\ 0.227EBV_{RGR}–0.021EBV_{RR} \end{array}$$

$$\begin{array}{l} PCS2=0.569EBV_{BW}-0.083EBV_{WW}-0.006EBV_{ADG}+0.045EBV_{KR}+0.071EBV_{GE}+\\ 0.083EBV_{RGR}+0.797EBV_{RR} \end{array}$$

where EBV is the estimated breeding value of animal, BW is birth weight, WW is weaning weight, ADG is pre-weaning weight gain, KR is Kleiber ratio, GE is growth efficiency, RGR is the relative growth rate, and RR is survival of kids.

## Discussion

### Genetic relationship of traits

Birth weight is under the control of different systematic and genetic factors (animal genes and maternal genes), particularly the contribution of maternal effect is high in the early and get reduced when the age of animals increased. This could affect the strength of correlation of birth weight with other traits. The correlation estimates of traits based on estimated breeding value suggest that considering birth weight or survival to weaning and one or some of the traits related to feed efficiency (Kleiber ratio, growth efficiency, and relative growth rate) could be important to improve other traits indirectly, although the indirect selection response is also determined by the magnitude of heritability estimate. Therefore, traits such as Kleiber ratio, growth efficiency, relative growth rate, weaning weight, and pre-weaning weight gain were the least important to explain the total variation. Including all these traits in the breeding objective of Boer crossbred goats may be insignificant. The magnitude of heritability estimates, genetic correlation, and difficulty of trait measurement could affect the selection of traits to be considered as goal traits. The (co)variance and heritability estimates for these traits were described in detail in previous studies [6,7,28]. The direct heritability estimate for birth weight, weaning weight, and pre-weaning weight gain was reasonable [6], which indicates the possibility of genetic improvement through selection. On the other hand, the heritability estimate for efficiency-related traits [7] and survival to weaning was low [28], and the expected genetic progress through selection could be slow due to low heritability estimates.

### Principal component and cluster analysis

The principal component analysis is the most commonly used multivariate tool used to reduce the dimensionality of large datasets in an interpretability way while minimizing information loss [29]. The estimate of Kaiser-Meyer-Olkin in this study is higher than the acceptable level (0.5) recommended by Kaiser [30], and higher than the value (0.6) recommended by Tabachnick and Fidell [31]. A measure of sampling adequacy and test of sphericity in this study indicates that the correlation matrix is not an identity matrix and provides sufficient justification for the validity of the principal component analysis of data [24]. The candidate traits loaded in the same component most likely share genomic sites responsible for their genetic regulation and the presence of a gene that exhibits multiple phenotypic expressions. The value of communality in this study is in line with the report of Valsalan et al. [24]. Communality indicates the proportion of variance in each variable that can be explained by the principal components. Communality values nearer one imply that the variation of a single variable is better explained by the extracted components. Thus, it shows that the variables have shared a lot of variances and the requirement to allow the PCA to classify them. However, the low communality value of BW suggests that BW explained little of the total variation in the factors according to Tesema et al. [32].

A clear distinction among traits could be important for farmers and breeders to consider important traits in the selection index and to improve other traits through indirect selection. According to Bodenmuller Filho et al. [33], the correlation is very high and positive if the angle between the variables (vectors) is close to zero; the correlation was also high but negative and will be more distant if the correlation is close to 180º; the variables were less correlated if the angle formed is about 90º. Thus, a high correlation was observed between weaning weight, pre-weaning weight gain, Kleiber ratio, relative growth rate, and growth efficiency as indicated in Fig 1. In addition, these traits were less correlated with birth weight and kids’ survival to weaning age.

The cluster analysis also shows the association among investigated traits clearly. Traits within the same cluster are regarded as more related to each other; thus, pre-weaning survival was associated with the birth weight of kids. Other studies [28,34–36] also noted a positive association of birth weight with kid survival, i.e., the risk of mortality is reduced with the increase of birth weight. Selection for all traits would be complex and may reduce genetic progress. Thus, selecting two traits (one from each group) could be enough and simplify the selection index. The remaining traits would be improved indirectly due to the correlation effect. For example, easily measurable traits such as birth weight could be used as indirect selection for kids’ survival. The trait classification using clusters method was align with the groupings observed in the principal components in the present study. In general, both the principal component and cluster analysis results indicated important biological relationships underlying the genetic relationships for traits important for crossbred goat breeding program.

### Selection indices

The best technique for genetic selection is the economic selection index. However, due to a need for economic data in livestock systems, estimating economic weights for each trait and derivimg economic selection index is difficult, paricularly in developing countries. The PCA allows for exploring the relationship between estimated breeding values, and it is an objective method to weight traits [14,18,20]. The PCA analyses weighted traits by considering each trait's contribution to the total variance in each principal component, which could be useful in developing countries where economic weights are not always available [20] due to absence of flock economic data. The larger absolute value of the PCS, the greater the relative importance of animals. The two PCS derived in this study allows capturing the main information in predicted breeding value for the investigated early growth, feed efficiency-related traits, and pre-weaning survival. Animals with a high PCS1 could enhance weaning weight, weight gain, Kleiber ratio, growth efficiency, and relative growth rate, whereas animals with a high PCS2 could improve birth weight and pre-weaning kid survival. The PCA-based selection indices are constructed more of based on the genetic merit of animals and correlation of traits. Thus, lack of considering the economic merit of traits could be the limitation of this approach. However, these indices are derived from easily measurable performance and pedigree data of animals, which makes easy to apply for selection of animals with the intervention of breeders. Therefore, these selection indices would be more important when economic weights for traits of interests are not available in time. This approach should be integrated into existing breeding programs to enhance selection accuracy, particularly in resource-limited areas where there is no detail recode of the flock, which help to estimate economic weight. For the traits found in the PCA-based selection indices, more investigation is required to determine exact economic weights. This will help refine the selection index and ensure that it aligns closely with the economic goals of the breeding program.

PCS1 seems in line with the objective of crossbreeding indigenous goats with improved Boer breed. The aim of crossing indigenous goats with exotic Boer goat was to improve the livelihood and income of producers by improving the growth performance and shortening the market age of goats. Nevertheless, working towards improving production only may not be sustainable and profitable if not integrated with fitness and efficiency-related traits of crossbred goats. Thus, the selection indices which incorporated production, efficiency-related trait and survival of kid help to improve the genetic progress towards the breeding goal, although the expected genetic progress reduced as the number of traits in the selection indices is increased. The selection of the most appropriate and specific selection index is determined by farmers’ breeding goals or breeding objectives.

## Conclusion

The correlation estimates indicates that considering birth weight or survival and one or some of the growth and efficiency-related traits could improve the other traits. Principal component and cluster analysis are useful tools for determining the total variation originating in a group of correlated traits and reducing the number of traits to be included in goat breeding programs’ selection index. Selection indices allow goat producers to make balanced selection decisions and identify the most profitable animal, taking into account each animal's relevant production and fitness traits. In this study, two principal components could explain the variation in the estimated breeding values of the analyzed traits. PC1 could be considered as new composite traits representing weaning weight, weight gain, Kleiber ratio, growth efficiency, and relative growth rate, whereas PC2 represents kids’ birth weight and survival. The choice of a specific selection index (principal component score) is determined by the breeding objectives that could be used in the genetic improvement programs. PCA-based selection indices could be used as an alternative approach when economic weights for traits of interests are not available to construct the economic selection index. However, further works should be done on refining the selection indices and validating them in independent datasets.

## Supporting information

S1 File Standardizedebv. (XLSX)
